# Activation of Einstein–Podolsky–Rosen steering sharing with unsharp nonlocal measurements

**DOI:** 10.1038/s41598-024-61649-4

**Published:** 2024-05-20

**Authors:** Xin-Hong Han, Tian Qian, Shan-Chuan Dong, Shuo Wang, Ya Xiao, Yong-Jian Gu

**Affiliations:** 1 College of Physics and Optoelectronic Engineering, Ocean University of China, Qingdao, 266100 People’s Republic of China; 2 College of Computer Science and Technology, Shandong University of Technology, Zibo, 255000 People’s Republic of China; 3 China Ship Research and Development Academy, Beijing, 100101 People’s Republic of China

**Keywords:** Quantum physics, Quantum information

## Abstract

Einstein–Podolsky–Rosen (EPR) steering is commonly shared among multiple observers by utilizing unsharp measurements. Nevertheless, their usage is restricted to local measurements and does not encompass all nonlocal measurement-based cases. In this work, a method for finding beneficial local measurement settings has been expanded to include nonlocal measurement cases. This method is applicable for any bipartite state and offers benefits even in scenarios with a high number of measurement settings. Using the Greenberger–Horne–Zeilinger state as an illustration, we show that employing unsharp nonlocal measurements can activate the phenomenon of steering sharing in contrast to using local measurements. Furthermore, our findings demonstrate that nonlocal measurements with unequal strength possess a greater activation capability compared to those with equal strength. Our activation method generates fresh concepts for conservation and recycling quantum resources.

## Introduction

In 1936, Schrödinger first proposed the concept of quantum steering as a response to the EPR paradox^1,2^. Many years later, Wiseman et al. point out the logic relation between EPR steering, nonlocality, and entanglement^3^. EPR steering^4^ logically sits between Bell nonlocality^5^ and quantum entanglement^2^ and exhibits a distinctive asymmetric property^6–11^, which describes the ability of one party, Alice, to nonlocally manipulate the state of another party, Bob, even if Bob does not trust Alice’s measurement apparatus. As an essential type of quantum resource, EPR steering has great applications in quantum key distribution^12,13^, subchannel discrimination^14^, asymmetric quantum network^15^, randomness generation^16,17^ and randomness certification^18^. Improving the utilization efficiency of EPR steering is of great importance, not only for fundamental quantum information science but also for applications in quantum communication.

To improve the utilization efficiency of EPR steering resources, researchers have relaxed the no-signaling condition^19^ and found that the steering of a single copy of the entangled states can be shared among multiple sequential observers either by unsharp measurements^19^ or standard projective measurements^20,21^. This method, known as steering sharing, has been extensively studied in bipartite systems^22,23^ and has also been used to investigate the reuse of genuine multipartite steering^24^.

Until now, all researches aimed at improving the efficiency of EPR steering has been restricted to local measurements^25–29^, In the case of local measurements, each observer measures the qubit in the hand. Mathematically, each observer’s measurement results can be obtained by measuring his or her respective reduced density matrices. However,nonlocal measurements mean operating instantaneous measurements on spacelike separate subsystems^30,31^. Indeed, nonlocal measurements between spatially separated observers cannot be accomplished through local measurements and classical communication. Thus, local measurement does not encompass all quantum information tasks that require nonlocal measurements, such as quantum teleportation. Nonlocal observables, however, are essential to quantum theory and can be found in everything from Bell inequalities and different postelection paradoxes to quantum error correction codes^30^. This raises some interesting questions: Is it possible to transform an unsteerable state into a steerable one by employing unsharp nonlocal measurements? If yes, how can we construct these beneficial nonlocal measurements? In particular, can unsharp nonlocal measurements still be more effective than unsharp local measurements in activating steering, even though each observer obtains the same classical measurement outcomes?

In this paper, we propose a steering sharing scenario using sequential unsharp nonlocal measurements. Focusing on the linear steering criterion for *n*-setting measurements, we extend a method that finds beneficial local measurement settings and apply it to nonlocal measurement scenarios. As an example, we consider the Greenberger–Horne–Zeilinger (GHZ) state in the case of two-setting measurements, and demonstrate that using unsharp nonlocal measurements can activate more steering sharing than using unsharp local measurements. We quantify the measurement strength ranges that can be used to activate the steering sharing and find that these ranges can be further extended by replacing equal-strength nonlocal measurements with unequal-strength ones. Our activation method produces new ideas for useful conservation and recycling of quantum resources.

The paper is organized as follows: In “Steering sharing using sequential unsharp nonlocal measurements”, we present the steering sharing scenario based on sequential unsharp nonlocal measurements. The method used to construct the beneficial nonlocal measurement settings is described in “Methods for finding beneficial unsharp nonlocal measurement settings”. An illustration of steering sharing activation is given in “Example of steering sharing activation”. Finally, we present the conclusion and some outlooks in “Discussion and conclusion”.

## Steering sharing using sequential unsharp nonlocal measurements

**Figure 1:**
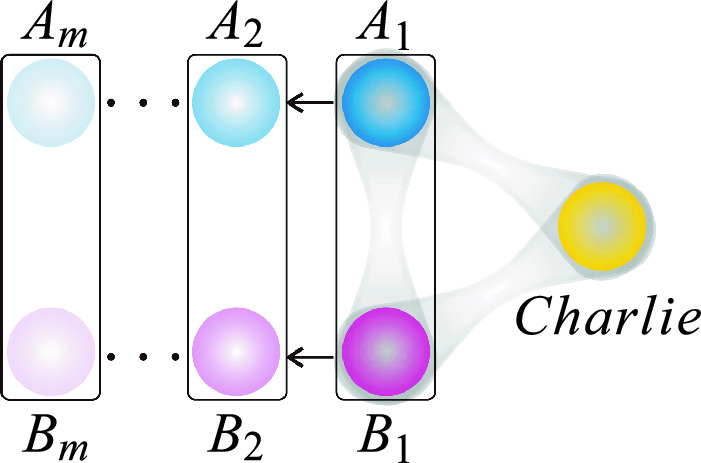
The scenario of steering sharing using sequential unsharp nonlocal measurements. A three-qubit state is initially shared between the spatially separated $A_1$, $B_1$ and Charlie. $A_1$ and $B_1$ perform nonlocal measurement on their qubits and transmit the post-measurement qubits to $A_2$ and $B_2$. This process is repeated until the last pair of $A_m$ and $B_m$ measure the qubits. Meanwhile, Charlie performs local measurements on his single qubit. The goal is for multiple pairs $\{A_i, B_i\}$, i.e., $\{A_1, B_1\}$, $\{A_2, B_2\},..., \{A_m, B_m\}$ to remotely steer the quantum state of Charlie simultaneously and independently.

Figure 1 illustrates a steering sharing scenario based on sequential unsharp nonlocal measurements. A three-qubit state $\rho _{ABC}$ is shared among Charlie and multiple pairs of observers, labeled as $A_i$ and $B_i$ where $i\in \{1,2,...,m\}$. The task of each pair of $A_i$ and $B_i$ is to remotely steer the quantum state of Charlie, simultaneously and independently. Charlie will be convinced by $A_i$ and $B_i$ if the correlation between their measurement outcomes cannot be explained by the LHV-LHS model. In our scenario, each pair of $A_i$ and $B_i$ (except the last $A_{m}$ and $B_{m}$) must perform unsharp measurements to generate strong correlations between them and Charlie to elude the LHV-LHS model, while preserving enough entanglement for the next pair of observers to achieve the same goal. Suppose the *k*-th nonlocal measurement setting of $A_i$ and $B_i$ is $\hat{\Pi }_{k}^{(i)}$ and the corresponding measurement strength is $\lambda _{k}^{(i)}$. And the *k*-th local sharp measurement setting of Charlie is denoted as $\hat{\Lambda }_k$. The success of the steering task among $A_{i}$, $B_{i}$ and Charlie can be tested by violating the bipartite *n*-setting linear steering inequality of the form^32^

1 $$\begin{aligned} S_n^{(i)}\!\equiv \!\frac{1}{n}\sum _{k=1}^n \lambda _{k}^{(i)}\langle \hat{\Pi }_{k}^{(i)}\otimes \hat{\Lambda }_{k}\rangle \!\le C_{n}. \end{aligned}$$

The bound $C_{n}=\underset{\Pi _{k}}{\textrm{max}}\{\lambda _{max}(\frac{1}{n}\sum _{k=1}^n \Pi _{k} \hat{\Lambda }_{k})\}$ is the maximum value of steering parameter $S_n^{(i)}$ if LHV-LHS model exists, where $\Pi _{k}\!\in \!\{++,+-,-+,--\}$ denotes a random variable, and $\lambda _{max}(\hat{U} )$ denotes the largest eigenvalue of $\hat{U}$. The expectation value $\langle \hat{\Pi }_{k}^{(i)}\otimes \hat{\Lambda }_{k}\rangle =\textrm{Tr}[\hat{\Pi }_{k}^{(i)}\otimes \hat{\Lambda }_{k} \rho _{ABC}^{(i)} ]$ is evaluated with respect to the average post-measurement states $\rho _{ABC}^{(i)}$ shared among $A_{i}$, $B_{i}$, and Charlie, which can be expressed as

2 $$\begin{aligned} \rho _{ABC}^{(i)}=\frac{1}{n}\!\sum _{k=1}^{n} \sum _{\mu ,\nu \!=\!-1,1}[(\!K^{(i)}_{\mu ,\nu |k}\otimes I_C )\rho _{ABC}^{(i-1)}({K^{(i)}_{\mu ,\nu |k}}\!^{\dagger }\otimes I_C)], \end{aligned}$$

where $\mu ,\nu \in \{+,-\}$ indicates the outcomes resulted from the $\hat{\Pi }_{k}^{(i)}$ of $A_{i}$ and $B_{i}$, $\hat{\Pi }_{\mu ,\nu |k}^{(i)}(\mu ,\nu \in \{+,-\})$ are the four elements of $\hat{\Pi }_{k}^{(i)}$. As unsharp nonlocal measurement $\hat{\Pi }_{k}^{(i)}$ is a particular class of positive operator valued measurement (POVM)^33^, the elements $\hat{\Pi }_{\mu ,\nu |k}^{(i)}$ can be implemented with corresponding Kraus operators as $\hat{\Pi }_{\mu ,\nu |k}^{(i)}\!=\!{K^{(i)}_{\mu ,\nu |k}}\!^{\dagger }K^{(i)}_{\mu ,\nu |k}$. And the four elements of $\hat{\Pi }_{k}^{(i)}$ satisfy that^25^

3 $$\begin{aligned} \begin{aligned}{}&\langle \psi |\hat{\Pi }_{\mu ,\nu |k}^{(i)}|\psi \rangle =\langle \psi |{K^{(i)}_{\mu ,\nu |k}}\!^{\dagger }K^{(i)}_{\mu ,\nu |k}|\psi \rangle =\langle \phi |\phi \rangle \ge 0,\\&\hat{\Pi }_{+,+|k}^{(i)}+\hat{\Pi }_{+,-|k}^{(i)}+\hat{\Pi }_{-,+|k}^{(i)}+\hat{\Pi }_{-,-|k}^{(i)}=I,\\&\hat{\Pi }_{+,+|k}^{(i)}-\hat{\Pi }_{+,-|k}^{(i)}-\hat{\Pi }_{-,+|k}^{(i)}+\hat{\Pi }_{-,-|k}^{(i)}=\lambda _{k}^{(i)}\hat{\Pi }_{k}^{(i)}. \end{aligned} \end{aligned}$$

Specifically, the elements $\hat{\Pi }_{\mu ,\nu |k}^{(i)}$ can be described as^22,25^

4 $$\begin{aligned} \begin{aligned}{}&\hat{\Pi }_{+,+|k}^{(i)}=\frac{1-\lambda _{k}^{(i)}}{4}I+\lambda _{k}^{(i)}E(\mu \!=\!+,\nu \!=\!+),\\&\hat{\Pi }_{+,-|k}^{(i)}=\frac{1-\lambda _{k}^{(i)}}{4}I+\lambda _{k}^{(i)}E(\mu \!=\!+,\nu \!=\!-),\\&\hat{\Pi }_{-,+|k}^{(i)}=\frac{1-\lambda _{k}^{(i)}}{4}I+\lambda _{k}^{(i)}E(\mu \!=\!-,\nu \!=\!+),\\&\hat{\Pi }_{-,-|k}^{(i)}=\frac{1-\lambda _{k}^{(i)}}{4}I+\lambda _{k}^{(i)}E(\mu \!=\!-,\nu \!=\!-), \end{aligned} \end{aligned}$$

where $E(\cdot )$ means the density matrix for corresponding eigenstate of nonlocal measurement $\hat{\Pi }_{k}^{(i)}$, *I* is the identity matrix.

To demonstrate the existence of steering ability among $A_{i}$, $B_{i}$ and Charlie, it is essential to ensure that their measurement settings $\{\hat{\Pi }_{k}^{(i)}, \hat{\Lambda }_{k}\}$ can achieve the better violation of the linear steering inequality Eq. (1).

## Methods for finding beneficial unsharp nonlocal measurement settings

Building on the previous method^34^, we aim to extend the method and find the beneficial unsharp nonlocal measurement settings $\{\hat{\Pi }_{k}^{(i)}, \hat{\Lambda }_{k}\}$ for detecting steering in the scenario depicted in Fig. 1. The specific steps are to first fix the measurement setting ${\hat{\Lambda }}_{k}$ in a certain direction, and then explore the corresponding measurement setting $\hat{\Pi }_{k}^{(i)}$ to maximize $\langle \hat{\Pi }_{k}^{(i)}\rangle$; then, change ${\hat{\Lambda }}_{k}$ to another direction and repeat the above process; finally, by searching $\hat{\Lambda }_{k}$ in the entire operator space, the measurement settings $\{\hat{\Pi }_{k}^{(i)}, {\hat{\Lambda }}_{k}\}$ that increase the difference between $S_n^{(i)}$ and $C_n$ can be obtained.

In the process, given the measurement setting for Charlie is fixed, we can straightforwardly calculate the corresponding beneficial measurement setting $\hat{\Pi }_{k}^{(i)}$ for $A_{i}$ and $B_{i}$ with the following two conditions: Firstly, the eigenvector list $\{e_{k}^{(i)}\}$ of $\hat{\Pi }_{k}^{(i)}$ is the same as the normalized conditional state $\widetilde{\rho }_{AB}^{(i)}$ of $A_{i}$ and $B_{i}$ after Charlie measures his qubit by $\hat{\Lambda }_k$. Secondly, the eigenvector list $\{\alpha _{k}^{(i)}\}$ of $\hat{\Pi }_{k}^{(i)}$ has the same order as the eigenvalue list $\{\beta _{k}^{(i)}\}$ of $\widetilde{\rho }_{AB}^{(i)}$^34^. And the situation is similar when the measurement setting $\hat{\Pi }_{k}^{(i)}$ of $A_{i}$ and $B_{i}$ is fixed. In fact, when the state $\rho _{ABC}^{(i)}$ coincides with the eigenstate of measurement operator $\{\hat{\Pi }_{k}^{(i)}\otimes \hat{\Lambda }_{k}\}$, the nonlocal measurement $\hat{\Pi }_{k}^{(i)}$ is beneficial.

Using the three-qubit GHZ state $\vert \textrm{GHZ}\rangle =(\vert 000\rangle +\vert 111\rangle )/\sqrt{2}$ as an example, we may apply the method mentioned above to acquire the beneficial nonlocal measurement settings, which can activate more EPR steering sharing. According to research^35^, the ideal measurement operator for GHZ state under local measurement is typically made up of a combination of $\sigma _{x}$ and $\sigma _{y}$. Therefore, here we first assume that Charlie’s measurement setting is $\hat{\Lambda }_1=\sigma _{x}$, then the elements $\hat{\Pi }_{\mu ,\nu |k}^{(i)}$ can be found using the method mentioned above, and Eq. (4) can be expressed as follows

5 $$\begin{aligned} \begin{aligned}{}&\hat{\Pi }_{+,+|1}^{(i)}=\frac{1-\lambda _{1}^{(i)}}{4}I+\frac{\lambda _{1}^{(i)}}{2}(|11\rangle -|00\rangle )(\langle 11|-\langle 00|),\\&\hat{\Pi }_{+,-|1}^{(i)}=\frac{1-\lambda _{1}^{(i)}}{4}I+\frac{\lambda _{1}^{(i)}}{2}(|11\rangle +|00\rangle )(\langle 11|+\langle 00|),\\&\hat{\Pi }_{-,+|1}^{(i)}=\frac{1-\lambda _{1}^{(i)}}{4}I+\frac{\lambda _{1}^{(i)}}{2}(|10\rangle -|01\rangle )(\langle 10|-\langle 01|),\\&\hat{\Pi }_{-,-|1}^{(i)}=\frac{1-\lambda _{1}^{(i)}}{4}I+\frac{\lambda _{1}^{(i)}}{2}(|10\rangle +|01\rangle )(\langle 10|+\langle 01|). \end{aligned} \end{aligned}$$

Moreover, when the measurement setting for Charlie is changed to $\hat{\Lambda }_2=\sigma _{y}$, the Eq. (4) will be rewritten as

6 $$\begin{aligned} \begin{aligned}{}&\hat{\Pi }_{+,+|2}^{(i)}=\frac{1-\lambda _{2}^{(i)}}{4}I+\frac{\lambda _{2}^{(i)}}{2}(|11\rangle -i|00\rangle )(\langle 11|+i\langle 00|),\\&\hat{\Pi }_{+,-|2}^{(i)}=\frac{1-\lambda _{2}^{(i)}}{4}I+\frac{\lambda _{2}^{(i)}}{2}(|11\rangle +i|00\rangle )(\langle 11|-i\langle 00|),\\&\hat{\Pi }_{-,+|2}^{(i)}=\frac{1-\lambda _{2}^{(i)}}{4}I+\frac{\lambda _{2}^{(i)}}{2}(|10\rangle +i|01\rangle )(\langle 10|-i\langle 01|),\\&\hat{\Pi }_{-,-|2}^{(i)}=\frac{1-\lambda _{2}^{(i)}}{4}I+\frac{\lambda _{2}^{(i)}}{2}(|10\rangle -i|01\rangle )(\langle 10|+i\langle 01|), \end{aligned} \end{aligned}$$

Thus far, the beneficial two-setting measurements for the GHZ state has been established, i.e. $\{\lambda _{1}^{(i)}\hat{\Pi }_{1}^{(i)}=\hat{\Pi }_{+,+|1}^{(i)}-\hat{\Pi }_{+,-|1}^{(i)}-\hat{\Pi }_{-,+|1}^{(i)}+\hat{\Pi }_{-,-|1}^{(i)}$, $\hat{\Lambda }_1=\sigma _{x}\}$ and $\{\lambda _{2}^{(i)}\hat{\Pi }_{2}^{(i)}=\hat{\Pi }_{+,+|2}^{(i)}-\hat{\Pi }_{+,-|2}^{(i)}-\hat{\Pi }_{-,+|2}^{(i)}+\hat{\Pi }_{-,-|2}^{(i)}$, $\hat{\Lambda }_2=\sigma _{y}\}$.

Similarly, we can always get nonlocal measurements of $A_{i}$ and $B_{i}$, provided we know the measurement direction of Charlie. By searching $\hat{\Lambda }_{k}$ in the entire operator space, the beneficial measurement settings $\{\hat{\Pi }_{k}^{(i)}, {\hat{\Lambda }}_{k}\}$ can be obtained. Furthermore, we will be able to activate more EPR steering sharing with the aid of these unsharp nonlocal measurements than we could with local ones.

**Figure 2:**
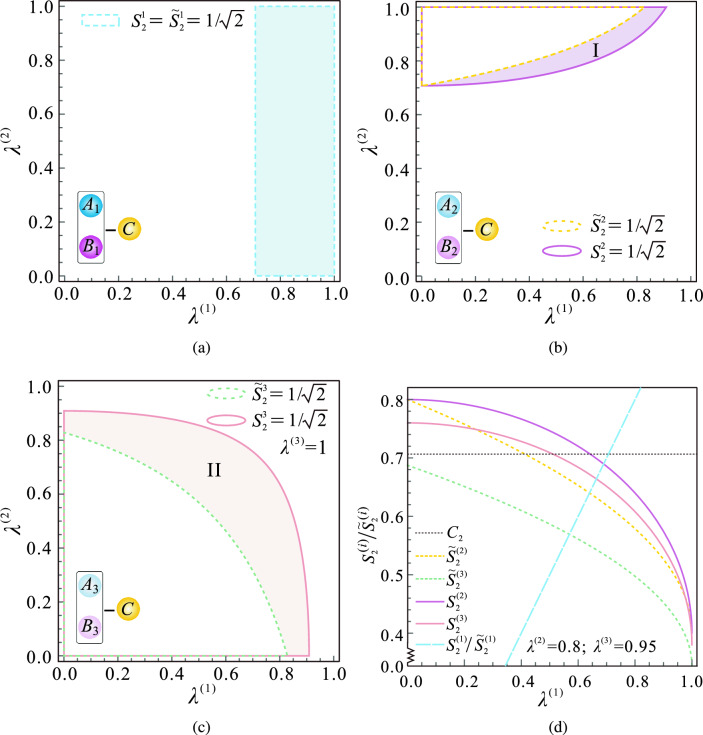
The comparison diagram illustrating the successful steering regions for unsharp nonlocal measurements versus unsharp local measurements. Specifically, (**a–c**) respectively show the successful steering regions for the first, second, and third pairs of $A_i$ and $B_i$. In each of these diagrams, the solid and dotted curves represent the boundaries of $S_2^{(i)}=1/\sqrt{2}$ and ${\widetilde{S}}_2^{(i)}=1/\sqrt{2}$, respectively. The regions within these boundaries indicate successful steering between the corresponding pairs of observers. Notably, the purple region (labeled as I) and the pink region (labeled as II) correspond to ${S_{2}^{(2)}\!>\!1\sqrt{2} \& {\widetilde{S}}_{2}^{(2)}\le 1\sqrt{2}}$ and ${S_{2}^{(3)}\!>\!1\sqrt{2} \& {\widetilde{S}}_{2}^{(3)}\le 1\sqrt{2}}$ respectively. (**d**) Steering parameters $S_2^{(i)}$ and ${\widetilde{S}}_2^{(i)}$ as a function of the measurement strength $\lambda ^{(1)}$ when $\lambda ^{(2)}=0.8$ and $\lambda ^{(3)}=0.95$.

## Example of steering sharing activation

In this section, we use the three-qubit GHZ state as an illustration to show how sequential unsharp nonlocal measurements can activate the sharing ability of steering that cannot be realized by sequential unsharp local measurements. To simplify the analysis, we focus on the case of two-setting measurements. The POVM elements of nonlocal measurement of $A_i$ and $B_i$ are displayed in Eq. (5) and Eq. (6) when Charlie’s measurement settings are $\hat{\Lambda }_1\!=\!\sigma _{x}, \hat{\Lambda }_2\!=\!\sigma _{y}$, respectively. Then the steering inequality Eq. (1) can be rewritten as

7 $$\begin{aligned} S_2^{(i)}\!=\!\frac{1}{2^i}\Big [\lambda _{2}^{(i)}\!\prod \limits _{1\le j\le i\!-\!1}\!(1\!+\!F_{\lambda _{1}^{(j)}})+\lambda _{1}^{(i)}\!\prod \limits _{1\le j\le i\!-\!1}\!(1\!+\!F_{\lambda _{2}^{(j)}})\Big ]\!\le \!C_{2}, \end{aligned}$$

where $F_{\lambda _{1}^{(j)}}=\sqrt{1-({\lambda _{1}^{(j)}})^2}$, $F_{\lambda _{2}^{(j)}}=\sqrt{1-({\lambda _{2}^{(j)}})^2}$. Given that $\{\Pi _{1}, \Pi _{2}\}\!\in \!\{++,+-,-+,--\}$ denote the random variables, then the classical bound $C_{2}=\underset{\Pi _{1},\Pi _{2}}{\textrm{max}}\{\lambda _{max}(\frac{1}{2}(\Pi _{1} \hat{\Lambda }_{1}+\Pi _{2} \hat{\Lambda }_{2}))\}\!=\!1/\sqrt{2}$ can be obtained by finding the maximum of 16 largest eigenvalues in Eq. (8) below.

8 $$\begin{aligned} \begin{aligned}{}&\{\!\Pi _{1}\!=\!++,\Pi _{2}\!=\!++\!\}, \{\!\Pi _{1}\!=\!++,\Pi _{2}\!=\!--\!\}, \{\!\Pi _{1}\!=\!--,\Pi _{2}\!=\!--\!\}, \{\!\Pi _{1}\!=\!--,\Pi _{2}\!=\!++\!\}\\&\quad : \lambda _{max}\left( \frac{1}{2}\left( \sigma _{x}+\sigma _{y}\right) \right) \!=\!1/\sqrt{2};\\&\{\!\Pi _{1}\!=\!++, \Pi _{2}\!=\!+-\!\}, \{\!\Pi _{1}\!=\!++, \Pi _{2}\!=\!-+\!\},\{\!\Pi _{1}\!=\!--, \Pi _{2}\!=\!+-\!\}, \{\!\Pi _{1}\!=\!--, \Pi _{2}\!=\!-+\!\}\\&\quad : \lambda _{max}\left( \frac{1}{2}\left( \sigma _{x}-\sigma _{y}\right) \right) \!=\!1/\sqrt{2};\\&\{\!\Pi _{1}\!=\!+-, \Pi _{2}\!=\!++\!\}, \{\!\Pi _{1}\!=\!-+, \Pi _{2}\!=\!++\!\},\{\!\Pi _{1}\!=\!+-, \Pi _{2}\!=\!--\!\}, \{\!\Pi _{1}\!=\!-+, \Pi _{2}\!=\!--\!\}\\&\quad : \lambda _{max}\left( \frac{1}{2}\left( -\sigma _{x}+\sigma _{y}\right) \right) \!=\!1/\sqrt{2};\\&\{\!\Pi _{1}\!=\!+-, \Pi _{2}\!=\!+-\!\}, \{\!\Pi _{1}\!=\!-+, \Pi _{2}\!=\!+-\!\},\{\!\Pi _{1}\!=\!+-, \Pi _{2}\!=\!-+\!\}, \{\!\Pi _{1}\!=\!-+, \Pi _{2}\!=\!-+\!\}\\&\quad : \lambda _{max}\left( \frac{1}{2}\left( -\sigma _{x}-\sigma _{y}\right) \right) \!=\!1/\sqrt{2}. \end{aligned} \end{aligned}$$

In comparison, when unsharp local measurements are used, the measurement settings for $A_i$, $B_i$, and Charlie would be $\{\hat{M}_{1}^{A_i}=\eta _{1}^{(i)} \sigma _y, \hat{M}_{1}^{B_i}= \gamma _{1}^{(i)} \sigma _y, \hat{M}_{1}^{C}=\sigma _x\}$ and $\{\hat{M}_{2}^{A_i}=\eta _{2}^{(i)}\sigma _y,\hat{M}_{2}^{B_i}=\gamma _{2}^{(i)}\sigma _x, \hat{M}_{2}^{C}=\sigma _y\}$. The corresponding steering parameter ${\widetilde{S}} _2^{(i)}$ can be expressed as

9 $$\begin{aligned} {\widetilde{S}} _2^{(i)}\!=\!\frac{1}{2^{i}}\Big [\lambda _{2}^{(i)}\!\prod \limits _{1\le j\le i\!-\!1}\!(1\!+\!F_{\gamma _{1}^{(j)}})+\lambda _{1}^{(i)}\!\prod \limits _{1\le j\le i\!-\!1}\!(1\!+\!F_{\gamma _{2}^{(j)}})\Big ]\le \!C_{2}, \end{aligned}$$

where $F_{\gamma _{1}^{(j)}}=\sqrt{1-({\gamma _{1}^{(j)}})^2}$, $F_{\gamma _{2}^{(j)}}=\sqrt{1-({\gamma _{2}^{(j)}})^2}$. And similar to the nonlocal measurement case abovementioned, the classical bound in the local measurement case can also be obtained as $C_{2}=1/\sqrt{2}$. The specific values of measurement strength parameters $\lambda _{k}^{(i)}$, $\eta _{k}^{(i)}$ and $\gamma _{k}^{(i)}$ should satisfy $\lambda _{k}^{(i)}=\eta _{k}^{(i)}*\gamma _{k}^{(i)}$, $k\in \{1,2\}$. It should be noted that since the GHZ state is an eigenstate of $\hat{M}_{k}^{A_i}\otimes \hat{M}_{k}^{B_i}\otimes \hat{M}_{k}^{C}$, these measurement settings are still optimal in the case of unsharp local measurements.

### The measurement settings with equal strength

We first investigate the activation of steering sharing when the strength of the two-setting nonlocal measurements used by $A_i$ and $B_i$ is equal, i.e., $\lambda ^{(i)}=\lambda _{1}^{(i)}=\lambda _{2}^{(i)}$, and the strength of two-setting local measurements used by each observer is equal, i.e., $\eta ^{(i)}=\eta _{1}^{(i)}=\eta _{2}^{(i)}$, $\gamma ^{(i)}\!=\!\gamma _{1}^{(i)}\!=\!\gamma _{2}^{(i)}$. The steering parameters in Eqs. (7) and  (9) can be respectively rewritten as $S_2^{(i)}=\frac{1}{2^{i-1}}\Big [\lambda ^{(i)}\prod \limits _{1\le j\le i\!-\!1}(1\!+\!\sqrt{1-({\lambda ^{(j)}})^2})\Big ]$ and ${\widetilde{S}} _2^{(i)}=\frac{1}{2^{i-1}}\Big [\lambda ^{(i)}\prod \limits _{1\le j\le i\!-\!1}(1\!+\!\sqrt{1-({\gamma ^{(j)}})^2})\Big ]$. Obviously, in order to satisfy the condition of $\lambda ^{(i)}=\eta ^{(i)}*\gamma ^{(i)}$, if $\lambda ^{(i)}<\gamma ^{(i)}$, then $S _2^{(i)}>{\widetilde{S}} _2^{(i)}$. In other words, more steering sharing can be discovered in the scenario of nonlocal measurements compared to that of local measurements. As a result, unsharp nonlocal product measurement can be used to activate the sharing ability of steering.

To clarify the effects of nonlocal measurements on activating steering sharing, we set $\eta ^{(i)} = \gamma ^{(i)} = \sqrt{\lambda ^{(i)}}$, where $i \in \{1, 2, 3\}$. Figure 2a–c show the steering regions for the first, second, and third pairs of $A_{i}$ and $B_{i}$, respectively. These regions are parameterized by the measurement strength $\lambda ^{(1)}$ and $\lambda ^{(2)}$. Obviously, the ranges of $\lambda ^{(1)}$ and $\lambda ^{(2)}$ that can be used to verify the existence of steering among $A_{1}$, $B_{1}$ and Charlie are the same in both unsharp nonlocal and local measurements. However, as the number of pairs of $A_{i}$ and $B_{i}$ increases, the ranges of $\lambda ^{(1)}$ and $\lambda ^{(2)}$ that satisfy $S _2^{i}>1\sqrt{2}$ are larger than the ranges that satisfy ${\widetilde{S}} _2^{i}>1\sqrt{2}$. For example, in Fig. 2b, c, the steering regions I (marked in purple) and II (marked in pink) can be activated with unsharp nonlocal measurements. This suggests that nonlocal measurement can activate steering sharing that is impossible to achieve through local measurement.

In Fig. 2d, we present the steering parameters $S_2^{(i)}$ and ${\widetilde{S}}_2^{(i)}$ ($i\in \{1,2,3\}$) varying with the measurement strength $\lambda ^{(1)}$. $S_2^{(1)}$ and ${\widetilde{S}}_2^{(1)}$ are represented by the same dashed blue line. $S_2^{(2)}$ and ${\widetilde{S}}_2^{(2)}$ are represented by the solid purple and dotted yellow lines, respectively. $S_2^{(3)}$ and ${\widetilde{S}}_2^{(3)}$ are represented by the solid pink and dotted green lines, respectively. Especially, when the measurement strength $\lambda ^{(1)}=0.5$, $S_2^{(2)}=0.75$ and is greater than $1\sqrt{2}$, while ${\widetilde{S}}_2^{(2)}=0.68$ and is less than $1\sqrt{2}$, Therefore, unsharp nonlocal measurements can activate more steering. When $\lambda ^{(1)}=0.4$, the amounts by which $S _2^{(2)}$ and ${\widetilde{S}}_2^{(2)}$ exceed the classical bound $1\sqrt{2}$ are 0.003 and 0.059, respectively. This means that steering can be easier to implement in experiments via unsharp nonlocal measurements. Additionally, it is clear that the values of ${\widetilde{S}}_2^{(3)}$ are consistently lower than the classical bound $C_2$, whereas $S_2^{(3)}$ has the potential to exceed $C_2$. And it is possible for $S_2^{(3)}$ to exceed ${\widetilde{S}}_2^{(2)}$. For example, when $\lambda _{1}=0.4$, ${\widetilde{S}}_2^{(2)}$ and ${\widetilde{S}}_2^{(3)}$ have values of 0.71 and 0.61, respectively, while $S_2^{(2)}$ and $S_2^{(3)}$ have values of 0.77 and 0.73, respectively. It is also evident that there are no values of $\lambda ^{(1)}$ that satisfy $S_{2}^{(1)}>1\sqrt{2}$, $S_{2}^{(2)}>1\sqrt{2}$, and $S_{2}^{(3)}>1\sqrt{2}$. This indicates that at most two pairs of $A_{i}$ and $B_{i}$ can share steering with Charlie simultaneously.

**Figure 3:**
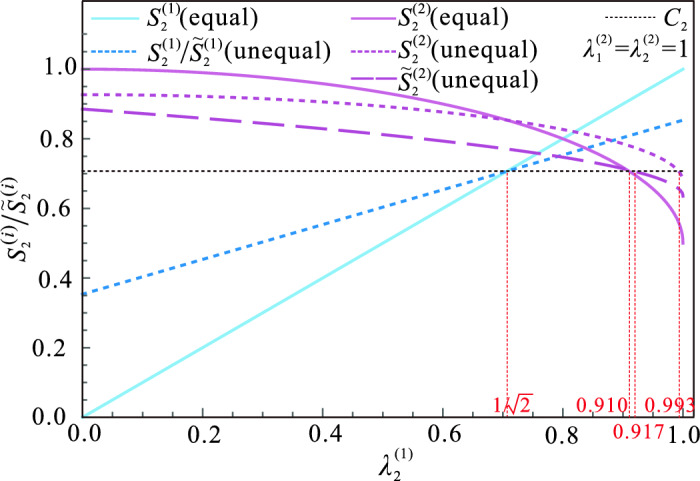
The steering parameters $S_2^{(i)}$ and ${\widetilde{S}}_2^{(i)}$ as functions of measurement strength $\lambda _2^{(1)}$. The solid line represents the situation when the measurement strengths are equal, while the dotted and dashed lines represent the situation when the measurement strengths are unequal. The blue and purple lines, respectively, indicate the steering parameters of $A_1$ and $B_1$, $A_2$ and $B_2$.

**Figure 4:**
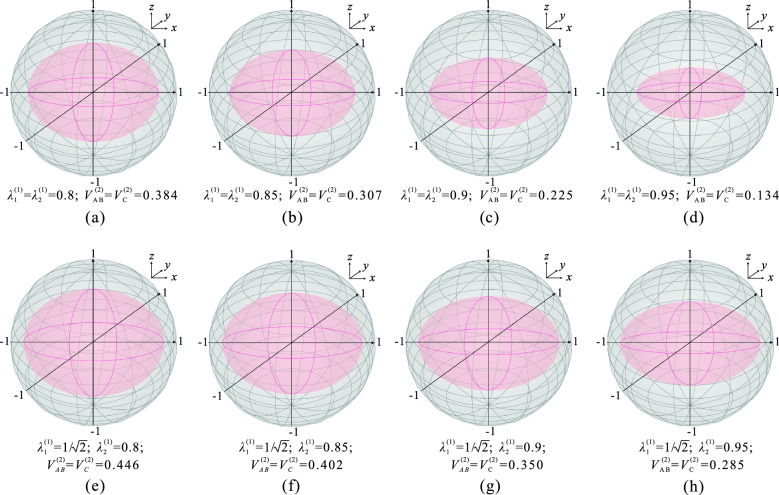
(**a–d)** The steering ellipsoids $\Omega _{AB}^{(2)}$ and $\Omega _{C}^{(2)}$ as the function of $\lambda _{1}^{(1)}$ and $\lambda _{2}^{(1)}$when $\lambda _{1}^{(1)}=\lambda _{2}^{(1)}$. (**e–h**) The steering ellipsoids $\Omega _{AB}^{(2)}$ and $\Omega _{C}^{(2)}$ as the function of $\lambda _{2}^{(1)}$ when $\lambda _{1}^{(1)}=1/\sqrt{2}$. The inner pink ellipsoids represent the steering ellipsoid, and the outer gray spheres represent the Bloch spheres.

### The measurement settings with unequal strength

Here, we relax the requirement that the strength of the two-setting nonlocal measurements used by each pair of $A_i$ and $B_i$ need to be equal, i.e., $\lambda _{1}^{(i)}\ne \lambda _{2}^{(i)}$. We also relax the requirement that the strength of the two-setting local measurements used by each $A_i$ and $B_i$ need to be equal, i.e., $\eta _{1}^{(i)}\ne \eta _{2}^{(i)}$, $\gamma _{1}^{(i)}\ne \gamma _{2}^{(i)}$. However, we still ensure that $\eta _k^{(i)} = \gamma _k^{(i)} = \sqrt{\lambda _k^{(i)}}$ is satisfied, where $k \in \{1, 2\}$. We find the maximum number of pairs of $A_i$ and $B_i$ that can simultaneously share steering with Charlie can not be increased by using measurements with unequal strength. Figure 3 illustrates the effect of measurement strength $\lambda _{2}^{(1)}$ on the steering parameters in three cases: (i) using unequal strength local measurements (dotted blue line for $A_1$ and $B_1$, dashed purple line for $A_2$ and $B_2$), (ii) using equal strength nonlocal measurements (solid blue line for $A_1$ and $B_1$, solid purple line for $A_2$ and $B_2$) and (iii) using unequal strength nonlocal measurements (dotted blue line for $A_1$ and $B_1$, dotted purple line for $A_2$ and $B_2$). It is evident that the steering parameters of the first and second pairs of $A_i$ and $B_i$ can exceed the classical bound at the same time when the measurement strength $\lambda _{2}^{(1)}$ is increased to $1/\sqrt{2}$ in all three cases. Additionally, the range of $\lambda _{2}^{(1)}$ that steering can be shared simultaneously among the first and second pair of $A_1$, $B_1$, $A_2$, $B_2$ and Charlie is $(1/\sqrt{2},0.917)$ and $(1/\sqrt{2},0.910)$ in case (i) and case (ii) respectively, which can be further extended to $(1/\sqrt{2},0.993)$ in case (iii). The results show that when $\lambda _{2}^{(1)}$ is in the range of (0.917, 0.993), the sharing of EPR steering can be further activated by using nonlocal measurements with unequal strength.

In addition, the quantum steering ellipsoid represents the states that the steering party can collapse the steered party to, considering all possible measurements performed on his subsystem^36^. To provide a more intuitive visualization of the distinction between steering sharing activation of equal and unequal strength nonlocal measurements, we also examined how the steering ellipsoids of $A_2$ and $B_2$, as well as steering ellipsoids of Charlie change as the measurement strengths $\lambda _{1}^{(1)}$ and $\lambda _{2}^{(1)}$ vary. However, since the steering ellipsoid is only available for two-qubit systems, we need to compress the three-qubit state into a two-qubit state. By defining $\vert 00\rangle \equiv \vert {\widetilde{0}} \rangle$ and $\vert 11\rangle \equiv \vert {\widetilde{1}}\rangle$, we can compress the state $\rho _{ABC}^{(i)}$ in terms of a two-qubit state.

Considering all possible measurements by $A_i$ and $B_i$, the state of Charlie can be steered to an ellipsoid $\Omega _{C}^{(i)}$, which is centered at $o_{C}^{(i)} = (\vec {n} - T\widetilde{m})/(1-|{\widetilde{m}}|^2)$. The orientation and the squared lengths of the ellipsoid’s semiaxes are given by the eigenvectors and eigenvalues of the ellipsoid matrix^36^

10 $$\begin{aligned} O_{C}^{(i)}=\dfrac{(T-\vec {n}\widetilde{m}^{\intercal })}{1-|{\widetilde{m}}|^2}\left( I+\dfrac{\widetilde{m}\widetilde{m}^{\intercal }}{1-|{\widetilde{m}}|^2 }\right) \left( T^{\intercal }-\widetilde{m}\vec {n}^{\intercal }\right) , \end{aligned}$$

where $\widetilde{m}$ and $\vec {n}$ are the Bloch vectors of the reduced states $\rho _{AB}^{(i)}$ and $\rho _{C}$ of $\rho _{ABC}^{(i)}$, *T* is the correlation matrix, and *I* is the identity operator. Similarly, the steering ellipsoid $\Omega _{AB}^{(i)}$ of $A_i$ and $B_i$ can be obtained by swapping the roles of Charlie with $A_i$ and $B_i$.

The volume of Charlie’s steering ellipsoids generated by the measurements of $A_i$ and $B_i$ can be written as^37^

11 $$\begin{aligned} V_{C}^{(i)}=\frac{|\det (T-{\widetilde{m}}{\vec {n}}^\top )|/(1-|{\widetilde{m}}{\vec {n}}|^2)^2}{4\pi /3}. \end{aligned}$$

Obviously, the volume of steering ellipsoids is normalized relative to the total volume of the Bloch sphere, which is $4\pi /3$^37^. Similarly, the volume of the ellipsoid of $A_i$ and $B_i$ ($V_{AB}^{(i)}$), as generated by Charlie’s measurements, can also be obtained. For the post-measurement state, the volumes $V_{AB}^{(2)}$ and $V_{C}^{(2)}$ are the same whether equal or unequal strength measurements are used because the steering ellipsoids $\Omega _{AB}^{(2)}$ and $\Omega _{C}^{(2)}$ are identical. The results of nonlocal measurements with equal strength are presented in the first row of Fig. 4, while those of nonlocal measurements with unequal strength are shown in the second row of Fig. 4. Clearly, with the increasing of $\lambda _{1}^{(1)}$ and $\lambda _{2}^{(1)}$, the steering ellipsoid contracts towards the sphere’s center along all three principal axes simultaneously using equal strength measurements. However, when fixing $\lambda _{1}^{(1)}$ at $1/\sqrt{2}$, it can be observed that with the increase of $\lambda _{1}^{(2)}$, the steering ellipsoid only contracts towards the center of the sphere along the *y* and *z* axes. The length of principal axe in the *x* direction is 0.854, remains constant. The principal axe shrinks in length in the *y* direction at the same rate as in the case of equal strength measurements. In the *z* direction, the principal axe shrinks in length at a rate that is approximately half as slow as it is when measured with equal strength. However, the volume reduction rate of the steering ellipsoid is usually slower when nonlocal measurements of unequal strength are employed.

## Discussion and conclusion

To summarize, we have presented a scheme to activate EPR steering sharing among multiple pairs of $A_i$ and $B_i$ as well as a single Charlie using unsharp nonlocal measurements. Interestingly, we have found that unsharp nonlocal measurements can be used to discover more steering sharing than their local counterparts, although they behave the same in terms of measurement outcome. Additionally, we have demonstrated that the steering sharing activation of nonlocal measurements can be further enhanced by replacing equal strength measurements with unequal strength measurements. Therefore, there is greater ability of unsharp nonlocal measurement to activate additional steering sharing in contrast to unsharp local measurement, generating novel ideas for recycling quantum resources.

There are several relevant open problems that still need to be addressed. Firstly, there requires a rigorous proof anout whether the steering ellipsoid geometry have any potential to help find beneficial nonlocal measurements and lower search complexity. Secondly, it would be interesting to optimize the nonlocal measurement strategy to increase the number of observers who can share steering beyond the two pairs of sequential observers achieved in our work. This may be possible to achieve by employing unsharp nonlocal measurements on both sides, allowing sequential observers to share classical information and use an adaptive strategy, or by adopting mutually biased measurements, which requires further investigation in the future. Finally, our method could potentially be applied to other types of quantum correlations, such as Bell nonlocality, quantum entanglement, quantum coherence, and quantum contextuality. This may promote the development of general relevant information protocols, such as quantum random access code, self-testing, and quantum randomness expansion.

## Data Availability

All data generated or analysed during this study are provided within the manuscript.
